# Effect of Dietary Crude Protein on Animal Performance, Blood Biochemistry Profile, Ruminal Fermentation Parameters and Carcass and Meat Quality of Heavy Fattening Assaf Lambs

**DOI:** 10.3390/ani10112177

**Published:** 2020-11-21

**Authors:** Cristina Saro, Javier Mateo, Irma Caro, Diego Eloy Carballo, Miguel Fernández, Carmen Valdés, Raúl Bodas, Francisco Javier Giráldez

**Affiliations:** 1 Instituto de Ganadería de Montaña, CSIC-Universidad de León, Finca Marzanas s/n, Grulleros, 24346 León, Spain; miguel.fernandez.gutierrez@csic.es (M.F.); carmen.valdes@unileon.es (C.V.); j.giraldez@eae.csic.es (F.J.G.); 2 Departamento de Producción Animal, Universidad de León, Campus Vegazana s/n, 24071 León, Spain; 3 Departamento de Higiene y Tecnología de los Alimentos, Universidad de León, Campus Vegazana s/n, 24071 León, Spain; jmato@unileon.es (J.M.); diegocarballo2@hotmail.com (D.E.C.); 4 Área de Nutrición y Bromatología, Facultad de Medicina, Universidad de Valladolid, Av. Ramón y Cajal 7, 47005 Valladolid, Spain; irma.caro@uva.es; 5 Instituto Tecnológico Agrario de Castilla y León, Av. Burgos, km 119, 47071 Valladolid, Spain; bodrodra@itacyl.es

**Keywords:** Assaf, feed efficiency, meat quality traits, protein

## Abstract

**Simple Summary:**

This study was designed to assess the optimal level of crude protein inclusion in the diet of heavy fattening Assaf lambs. Our results suggested that levels of crude protein between 134 and 173 g/kg DM (dry matter) influence the dry matter intake and performance of lambs, but no effects were observed on rumen function, animal health, or carcass and meat quality. To maximize growth performance, a minimum level of crude protein of 157 g/kg dry matter is needed, but protein content beyond that level does not improve meat quality and could worsen profitability.

**Abstract:**

Thirty Assaf male lambs (30 ± 1.9 kg of body weight) were allocated to three groups fed diets differing in their crude protein (CP) contents (low protein (LP), 134 g CP/kg dry matter (DM); medium protein (MP), 157 g CP/kg DM; and high protein (HP), 173 g CP/kg DM) to test the effect of dietary protein content on animal performance, rumen function, animal health, and carcass and meat quality. Feed intake was recorded daily, and animals were weighed every second week. Lambs were blood-sampled to determine their acid–base status and biochemical profile. After 70 days of trial, lambs were slaughtered, and the ruminal content was collected to assess ruminal fermentation. Finally, carcass and meat quality were evaluated. Dry matter intake and average daily gain increased (*p* < 0.05) when increasing the level of dietary CP. There were not significant differences (*p* > 0.05) in the evaluated parameters in the rumen fluid of lambs. There were not significant differences in carcass or meat quality (*p* > 0.05) and in those parameters related to blood acid–base status. Several biochemical parameters showed differences depending on diet CP level (urea, protein, albumin, glucose, and calcium; *p* < 0.05). Feeding costs calculated in relation to cold carcass weight decreased when dietary CP decreased. The results suggested that a dietary protein content greater than 157 g/kg DM would be required to maximize growth performance in Assaf male fattening lambs under 50 kg of body weight. However, a protein content beyond that level was not found to improve either carcass or meat quality and could worsen profitability.

## 1. Introduction

The Assaf breed has successfully spread in the Mediterranean area during the last four decades, and it is currently the main sheep breed in intensive dairy production systems in several countries [1,2,3]. In these systems, milk sales represent the main income component, and most male lambs are sold as milk-fed lambs [4,5]. Nevertheless, heavy fattening male lamb production is increasing in response to growing demand from African countries. It represents an important opportunity to compensate, in the short term, for seasonal fluctuations in local demand and, in the long term, for the reduction in red meat consumption in developed countries [6].

In practice, the feeding of fattening lambs very much depends on protein supplements since dietary protein content could affect feed intake, animal growth, and carcass and meat characteristics [7,8]. Nevertheless, protein over-feeding in ruminant diets projects a negative image of the production system because of both the environmental cost and the competition for these resources with humans and other livestock species that are more efficient than ruminants [9]. Moreover, Europe is dependent on overseas land for soybean meal, the source of protein most used in livestock feeding.

Different feeding strategies could be used to reduce protein over-feeding, but the first option should be to accurately meet animal requirements to avoid over-feeding protein. Most studies carried out in heavy fattening Assaf lambs have used diets with 17–20% of crude protein (CP; dry matter (DM) basis) [10,11,12,13,14]. However, this protein content is greater than that estimated as optimum by different feeding systems [15,16]. Likewise, Abo Omar and Naser [17] did not observe differences in either feed intake or average daily gain (ADG) in Assaf lambs fed diets containing 140 or 180 g of CP/kg DM. On the contrary, Fernandez et al. [18] reported differences in ADG in Assaf male lambs fed diets with 136 or 205 g CP/kg DM, and Rodríguez et al. [19] observed similar results when comparing the animal performance of light fattening Assaf lambs fed a conventional diet (166 g CP/kg DM) or raised on a free-choice feeding system where animals selected a diet containing 230 g CP/kg DM. In addition to these controversial results, most of these studies were focused on animal performance, ignoring effects on meat quality. Therefore, there is a need to establish the optimal level of protein in male Assaf lambs while considering its effect on animal performance and meat quality. It is important to note that most published studies regarding meat quality in Assaf lambs have been carried out with milk-fed and light fattening lambs [19,20], and, to the best of our knowledge, the scientific information published on heavy fattening meat Assaf lambs is scarce [14].

The objective of the present experiment was to evaluate the effect of dietary protein content on animal performance, feeding costs, ruminal fermentation pattern, blood acid–base status, biochemical profile, and carcass and meat quality in heavy fattening male Assaf lambs.

## 2. Materials and Methods

Experimental conditions and handling practices followed the recommendations of the Directive 2010/63/EU of the European Parliament and were evaluated and approved by the CSIC Animal Experimentation Committee (Protocol number 100102/2017-4).

### 2.1. Animals and Diets

Thirty male Assaf lambs were distributed in three experimental groups, equilibrated for weight (30 ± 1.9 kg) and age (89 ± 0.8 days), and they were randomly allocated to one of the three experimental diets differing in CP contents (134, 157, and 173 g of CP/kg DM for low protein (LP), medium protein (MP), and high protein (HP) diets, respectively). The ingredients and chemical composition of the experimental diets are shown in Table 1.

Animals were individually penned during the whole experimental period (70 days) and fed the corresponding experimental diet ad libitum. Lambs had free access to fresh water and were able to see and hear the other animals.

### 2.2. Experimental Procedure

Feed intake was recorded daily, with the amount offered adjusted to allow for refusals of a minimum of 10%. Samples of the feed offered and refusals for each animal were collected every day, and a composited sample per week was stored for subsequent analyses.

Body weight (BW) was recorded every 2 weeks before daily feeding. On days 0, 35, and 70 before morning feeding and after having removed the refusals, a blood sample was taken by jugular venipuncture into lithium heparin tubes, placed in ice, and then centrifuged at 3520× *g* for 20 min at 4 °C. Then, plasma samples were frozen at −20 °C until the analysis of the biochemical profile. On days 35 and 70, an extra blood sample was obtained, and samples were assayed for acid–base parameters at approximately 1 h after collection.

On day 71, all animals were slaughtered. After 8 h without feed, lambs were weighed, stunned, slaughtered by exsanguination from the jugular vein, eviscerated, and skinned. In 4 representative lambs of each group, the rumen was removed, its content was mixed, and then it was straightened through 4 layers of cheesecloth. Then, pH was measured, 0.8 mL of fluid were added to 0.5 mL of deproteinizing solution (20 g/L metaphosphoric acid and 0.6 g/L of crotonic acid) for volatile fatty acid (VFA) determination, and 4 mL were added to 20 µL of 20% H_2_SO_4_ (vol/vol) for ammonia-N analysis. The rest of ruminal fluid was used for the in vitro trial that is described in Section 2.5.

### 2.3. Carcass and Meat Characteristics

Carcass weight was recorded immediately after slaughter and after refrigeration at 4 °C for 24 h (cold carcass weight—CCW) in order to calculate chilling losses and dressing percentage. At 24 h, pH was measured on the longissimus thoracis muscle at the level of the 6th rib using a pH meter equipped with a penetrating electrode and a temperature probe (Metrohm, Switzerland). Subcutaneous fat color was determined using the CIELAB system and by calculating the chroma and hue values from the L* (lightness), a* (redness), and b* (yellowness) coordinates [21]. Color measurements were carried out in duplicate on the subcutaneous fat in the lumbar region near the tail base using a Minolta CM-2002 chroma meter (Konica-Minolta Sensing, Osaka, Japan) operating with the D65 illuminant, SCI mode, with a 10° visual angle, an 11 mm aperture for illumination, and an 8 mm aperture for measurement. Carcass internal length (L), chest width (Th), and pelvic limb length (F) were measured, and the compactness index of carcass (CIC—CCW/carcass internal length) was calculated [22].

The left sides of the carcasses were divided into commercial cuts (legs, foreribs, loin, shoulder, breast, neck, and tail), as described by Colomer-Rocher et al. [23], and these were weighed. After jointing, a transversal cut of the loin just after the 13th rib was carried out, and subcutaneous fat depth and l. lumborum width and depth were measured with a caliper [23]. Afterwards, the l. thoracis and l. lumborum muscles were removed from the corresponding joints for each half carcass and weighed together, and the weight percentage of the muscles in the half-carcass was calculated as 100 × (weight of l. thoracis and l. lumborum muscles/sum of the commercial cut weights). Leg tissue composition was determined by following the method of Fisher and de Boer [24].

Two 2.5 cm thick slices from the distal end of each l. thoracis muscle were cut and placed on a polypropylene tray, which was wrapped with polyvinylchloride cling film and refrigerated (4 °C) in darkness. At days 0 (2 h after storage), 5, and 10 of storage, the tray was unwrapped, the slices were weighed, and the color was determined in duplicate on the upper surface of the slices following the procedure previously described for the color of subcutaneous fat. Furthermore, in order to assess meat color stability during refrigerated storage, the following ratios of reflectance were calculated: 630 and 580 nm (R_630_/R_580_), 610 and 525 nm (R_610_/R_525_), and 572 nm and 525 nm (R_572_/R_525_) [25]. A third 2.5-cm thick slice was cut from the distal end of the l. thoracis muscle, and this slice was packaged under vacuum, cooked in water at 80 °C for 40 min, and cooled. Half of the slice was immediately analyzed for thiobarbituric acid reactive substances (TBARS), and the other half was wrapped into polyvinylchloride cling film, refrigerated (4 °C) for 48 h, and then analyzed for TBARS. The rest of the l. thoracis was used for chemical (proximate composition) analysis.

Cooking losses and meat hardness (shear force) were determined using the muscle l. lumborum, both at 24 h postmortem (day 0) and after 5 additional days of refrigerated (4 °C) storage. The muscle from the half carcass was transversely cut to obtain two similar portions (distal and caudal) that were randomly assigned to the day 0 or day 5 groups. The day 0 muscle portions were immediately weighed, vacuum-packaged and cooked in a water bath at 80 °C for 40 min, then cooled with tap water, removed from the packaging bag, dried on its surface with filter paper, and weighed again. Water loss was then determined as the difference in weight between cooked and raw meat, and it is expressed as percentage of raw meat. The day 5 portions were weighed and refrigerated for 5 days under the same conditions as described for the l. thoracis (color determination) before being weighed, vacuum-packaged, cooked, cooled, and weighed again. Prism-shaped (3 × 1 × 1 cm) samples from the cooked muscle portions (3–4 prisms per portion) were obtained and subjected to Warner–Bratzler shear force analysis according to Santos et al. [26].

### 2.4. In Vitro Fermentation

An in vitro trial was carried out to compare rumen fermentation pattern using starch and neutral detergent fiber (NDF—obtained from barley straw) as substrates. Rumen fluid was mixed with the culture medium, as described by Goering and Van Soest [27], in a 1:4 proportion. Incubations were performed in 120 mL serum bottles in which 300 mg of DM substrate were weighed. Rumen fluid from each lamb was used as a separate inoculum. Two bottles per substrate and animal and blanks were included in the incubation trial. After anaerobically dispensing 30 mL of the diluted rumen fluid, each bottle was sealed with rubber stoppers and aluminum seals, and all the bottles were placed in an incubator at 39 °C. At the end of the 24 h incubation, the total gas production was determined using a pressure transducer (Delta Ohm DTP704-2BGI, Herter Instrument SL, Barcelona, Spain) and a calibrated syringe, bottles were swirled in ice to stop fermentation, and then the bottles were opened to measure pH in the incubation medium. Samples for ammonia-N and VFA analysis were taken as described for samples taken at slaughter.

### 2.5. Protein Degradability

Protein degradability was determined in sacco using three ruminally fistulated Assaf ewes (68 ± 3.7 kg) that were fed the HP diet ad libitum. Nylon bags (125 × 100 mm size with 45 µm-sized mesh), containing 7 g of the corresponding diet that were ground to pass a 4 mm screen, were incubated in the rumen for 2, 6, 12, 24, and 48 h. Three bags (one per animal) were used for each time interval and diet. After incubation, bags were removed, rinsed slightly with tap water, and frozen for at least 5 days at −30 °C. Finally, all bags, including 2 extra bags per diet to estimate disappearance at zero time, were machine-rinsed using a cold water program for 30 min. Dried bags were weighed, residues were ground to pass a 1-mm screen, and the CP content of each was determined.

### 2.6. Chemical Analysis

The DM content of feeds, refusals, and in sacco residue samples were determined according to ISO 6496:1999 [28]. Feed samples were analyzed for ash (ISO 5984:2002, [29]) and protein (ISO 5983:2009, [30]). Crude fat was determined by the Ankom fiber bag technique [31]. aNDFom (neutral detergent fiber expressed without residual ash) and ADFom (acid detergent fiber expressed without residual ash) were analyzed as described by Van Soest et al. [32] using a fiber analyzer (Ankom Technology Corp., Macedon, NY, USA). Sodium sulfite and heat-stable amylase were used in the analysis of aNDFom. Ruminal fluid samples were analyzed for ammonia and VFA concentration, as previously described by Carro et al. [33].

Muscle samples were trimmed to eliminate connective tissue, homogenized, and freeze-dried to determine dry matter content. Afterwards, l. thoracis samples were analyzed for ash (AOAC official method 920.153), CP (AOAC official method 981.10), and fat (AOAC official method 960.39) contents. TBARS were determined following the method of Nam and Ahn (2003). Blood samples were assayed in a VetStat blood gas and electrolytes analyzer (Idexx, Barcelona, Spain) for pH, CO_2_ pressure (pCO_2_), total CO_2_ (tCO_2_), bicarbonate, and other parameters indicative of acid–base status, such as anion gap, Na, K, and Cl concentrations. Frozen plasma samples were defrosted overnight at 4 °C, and aspartate aminotransferase (AST), alanine aminotransferase (ALT), total cholesterol, glucose, triglycerides, lactate, calcium, phosphorus, urea, creatinine, total protein, and albumin contents were determined with an ILAB 650 biochemical profile autoanalyzer (Instrumentation Laboratory, Lexington, MA, USA).

### 2.7. Calculations

Protein degradability (dg) values were fitted with the model described by Orskov and McDonald [34]: $dg=a+b*\left(1-e^{\left(-c*t\right)}\right)$. The potential rumen-degradable protein (RDP) content of the experimental diets was calculated from the values estimated from the model (*a + b*) for each feed. The effective rumen degradation of CP was calculated following the method of AFRC [16] while assuming a rumen outflow rate (*k*p) of 0.083 h^−1^.

The ADG (g/d) was estimated as the regression coefficient (slope) of body weight against time, and the feed-to-gain ratio was obtained by dividing the dry matter intake (DMI) by the estimated ADG. Residual feed intake was calculated as described by Koch et al. [35].

### 2.8. Statistical Analysis

Degradability parameters a, b, and c were estimated by an iterative least squares procedure using the NLIN procedure of SAS (SAS Inst. Inc., Cary, NC, USA).

The data of the feed intake, ADG, feed efficiency, carcass characteristics, in vivo rumen fermentation, and tissue and chemical composition of the meat were analyzed using the MIXED procedure of SAS (SAS Inst. Inc., USA). The model included the fixed effect of the diet and the animal nested within the diet as residual error. The initial BW was also included as a covariate in the model, but it was removed because the effect was mainly non-significant (*p* > 0.05). Orthogonal polynomial contrasts were used for linear and quadratic trend comparisons.

Data from in vitro trial were averaged before statistical analysis (two values per animal), and they were analyzed with the above-described statistical model.

Data from blood gases and biochemical parameters, texture, cooking losses, and color of the meat were analyzed as a repeated measures model using the MIXED procedure of SAS. This procedure included the fixed effects of the diet, the experimental day, and their interactions, and the random effect of animal nested to the diet was used as the error to test the diet effect. The mean square of the day × animal (diet) interaction was used as the residual error to test the effects of the day and the interaction between the diet and the day. Different covariance matrixes were evaluated on the basis of Schwarz’s Bayesian information criteria. Plasma values at day 0 were used as a covariate, and they were removed from the model when their effect was not significant (*p* > 0.05). The level of significance was determined at *p* < 0.05, and means were separated using the least significant difference procedure.

## 3. Results

### 3.1. Feed Intake, Animal Performance and Fermentation Pattern

The DMI was not affected (*p* > 0.05) by dietary protein level, although when linear and quadratic effects were evaluated, the former was significant (*p* = 0.0422). The average daily gain and the feed-to-gain ratio increased (*p* < 0.01) and decreased (*p* < 0.01), respectively, with increasing levels of dietary protein. Nevertheless, the residual feed intake was unaffected by dietary treatments (*p* > 0.05, Table 2).

Table 3 shows the values of the in vivo and in vitro ruminal fermentation parameters. There were no differences (*p* > 0.05) among dietary treatments, either in the pH and ammonia concentration or in the total VFA concentration and the proportions of the individual VFAs in the ruminal fluid samples taken at slaughter. However, the HP group showed higher (*p* < 0.05) values of total VFA concentration and lower (*p* < 0.051) values of acetate proportion than the LP and MP groups when NDF from straw was used as the substrate in the in vitro fermentation assay. Only the ammonia concentration and the proportion of valerate plus caproate were significantly (*p* < 0.05) affected by dietary treatments when starch was used as the substrate, the values being higher in the HP group than in the LP group.

### 3.2. Blood Acid–Base Status and Metabolic Profile

Table 4 shows the values of parameters related to the acid–base status of the lambs, as well as their plasma biochemical profile. None of the parameters related to acid–base status were affected (*p* > 0.05) by dietary treatments. However, sampling day had a significant effect (*p* < 0.05) on blood tCO_2_, HCO_3_^−^, Na, K, and Cl concentration, the lowest values being observed at the end of the experimental period (day 70), and on anion gap (*p* < 0.05), with highest values on day 70.

The plasma concentrations of protein, albumin, glucose, and Ca were affected (*p* < 0.05) by dietary treatments, with LP lambs showing lower values than HP lambs. Lambs from the MP group showed intermediate values that were significantly (*p* < 0.05) different from LP values for albumin and calcium. The LP and MP groups had the lowest and highest urea concentration (*p* < 0.05), respectively, with HP lambs showing intermediate values. Concerning the sampling day, it had effect on all the parameters except for AST and lactate. The values were higher (*p* < 0.05) on day 35 than on day 70 for ALT and triglycerides, and the opposite was observed for the rest of the parameters.

### 3.3. Carcass and Meat Characteristics

The mean values of the carcass characteristics are displayed in Table 5. The effect of dietary protein level was not significant (*p* > 0.05) for any of the recorded quality traits, although a linear effect was observed for CCW (*p* linear = 0.0402).

The effect of dietary treatment by storage time interaction was not significant (*p* > 0.05) for any of the meat quality characteristics, and, hence, only the mean values for main factors are shown in Table 6. Dietary protein did not affect muscle composition, color, cooking losses, hardness, or lipid oxidative stability, as assessed by TBARS determination in cooked meat. Storage time in raw meat affected the color values related with meat discoloration, i.e., a* and a*/b* and the three reflectance ratios [25]. In cooked meat, storage time increased cooking losses and decreased shear force. The TBARS of meat just after cooking were below 0.1 mg of malondialdehyde per kg of meat; therefore, the increments in TBARS concentration during the two-day refrigerated storage were equivalent to the TBARS levels shown by the stored meat.

### 3.4. Feeding Costs

The feeding costs per kg of ADG were 1.90, 1.98, and 2.14 €/kg for the HP, MP, and LP groups, respectively. However, the feeding cost decreased as protein content decreased when it was expressed in relation to CCW (1.51, 1.48, and 1.42 €/kg CCW for the HP, MP, and LP groups, respectively).

## 4. Discussion

### 4.1. Feed Intake, Animal Performance and Ruminal Fermentation Pattern

There are now considerable experimental data supporting the role of dietary characteristics on feed intake control in ruminants, with energy density and protein content being the major dietary factors involved when high concentrate diets are used. However, while feed intake increases when energy density decreases [36,37], ad libitum fed fattening lambs do not show a compensatory intake when protein content decreases. In fact, most studies have reported no effect [8,17,37] or a decrease in feed intake as dietary protein content decreases [18,38,39], depending on different factors. In concordance to the latter cited studies, in this study, LP lambs showed the lowest values for feed intake and ADG.

Frequently, the interaction between feed intake and dietary protein content in diets for ruminants is related to changes in ruminal fermentation, with the fibrolytic microorganisms and, hence, the fiber digestibility being negatively affected by N underfeeding [40,41]. Several studies have reported a positive correlation between CP intake and both ruminal pH and ammonia concentration [42,43], although, in agreement with our results, these relationships have not always been observed [44,45]. Our samples were taken post-slaughter after 8 h of fasting, and this could explain the lack of effect of dietary protein level on ruminal ammonia concentration. In fact, in the in vitro assay, ammonia-N concentration was greater in the HP lambs than in the LP lambs. It must be pointed out that, in the three experimental groups, the in vivo concentration of N-ammonia at slaughter was greater than the minimal required for optimizing microbial growth [46], and it is not expected that lower concentrations occurred throughout the day. Nevertheless, it has been reported that microbiota composition shifts in response to dietary protein supply, cellulolytic bacteria growth, and, hence, fiber digestibility, being stimulated by amino acid supply [44,47]. This effect could explain the reduction of in vitro VFA production in the LP lambs in comparison to the HP lambs when NDF from straw was used as a substrate. This reduction, however, was not accompanied by a reduction in the proportion of acetate, as would have been expected [44].

Regardless the effect of dietary protein on ruminal fermentation, it has been confirmed that animals have a limited capacity to store a relative excess of energy as fat or to dissipate it as heat, which would limit feed intake when low protein/high energy diets are used [48,49]. In fact, no differences were observed between the LP and HP lambs in renal-pelvic fat, leg tissue composition, and intramuscular fat content, suggesting that fat deposition is limited in LP lambs. On the other hand, several studies have concluded that increasing protein content to 14–15% DM does not improve feed intake and growth performance in fattening lambs [8,17,37,50]. Nevertheless, in the present study, the ADG and feed-to-gain ratio were improved when CP content increased from 156 to 173 g/kg DM. It has been suggested that the effect of energy level is more important than the protein level only when high-energy diets are supplied [37]. However, these results may not be applied to the Assaf breed. Actually, Rodríguez et al. [19] reported that male light fattening Assaf lambs raised in a free-choice feeding systems selected high protein diets (23% of CP) and showed feed intake (885 vs. 759 g DM/day) and ADG (371 vs. 272 g/day) values greater than lambs consuming a high-concentrate conventional diet (16% of CP). Likewise, Rodríguez et al. [51] observed that a dietary protein content of 16.6% (on a dry matter basis) allowed male Assaf × Merino crossbreed lambs to achieve high growth rates, but it was not enough for male pure Assaf lambs. These results corroborate that the optimal protein content for male Assaf lambs is greater than 16%.

### 4.2. Blood Acid–Base Status and Biochemical Profile

Neither clinical signs of acute ruminal acidosis (feed intake depression, and diarrhea) nor laminitis were observed in any of the experimental animals, which was in concordance with the normal plasma lactate values that were recorded [52]. Ruminal pH values were also within a normal range (6.6 ± 0.35), but they were recorded after 8 h of fasting, and it is known that ruminal pH increases during fasting in ruminants [53]. In fact, the mean values of blood gas parameters and the anion gap were in agreement with those reported for fattening lambs with subacute ruminal acidosis [54].

It is known that ammonia released from the ruminal degradation of dietary or endogenous urea contributes to the maintenance of physiological ruminal pH. In the current study, plasma urea concentration was lower in the LP group than in the other experimental groups, which indicated a lower absorption of ruminal N-NH_3_ and urea synthesis in the liver. However, no differences among treatments were observed in blood acid–base parameters, suggesting that other factors, such as differences in feed intake or in endogenous urea could have counteracted the reduction in the buffer effect of the dietary N.

The plasma levels of hepatic enzymes, albumin, protein, creatinine, cholesterol, triglycerides, glucose, Ca, and P were within the normal values reported for ovine [55]. Despite that, the plasma levels of protein, albumin, glucose, and Ca increased as dietary protein increased. Albumin is the most abundant of circulating proteins, and it has been reported that albumin synthesis and catabolism (and hence its plasma concentration) are modulated by dietary protein intake [56,57]. In addition to other physiological roles, albumin provides amino acids to support peripheral tissue anabolism when the dietary protein supply is not enough to cover requirements [57]. Protein requirements are very high in growing ruminants and, as has been discussed above, the lower ADG in the LP group suggests a limiting protein supply that was compatible with the lower but physiological normal values of plasma albumin.

The difference in the plasma concentrations of glucose between the LP lambs and the other two groups was probably due to a lower dry matter intake, as the MP and HP lambs showed similar values of plasma glucose and feed intake. Propionate is the major substrate for gluconeogenesis in ruminants, and a reduction in propionate supply would be expected as dry matter and carbohydrate intakes decrease. In fact, glycemia does not seem to be affected by dietary protein intake when carbohydrate intake does not change [42,58].

### 4.3. Carcass and Meat Characteristics, and Feeding Costs

The mean values of carcass weight and dressing percentage were within the range of values reported for male Assaf lambs of similar BW [10,12]. To the best of our knowledge, there is not published information on the morphological carcass characteristics of heavy fattening male Assaf lambs, but they were within the range of values reported for other fat-tailed sheep breeds slaughtered at similar BWs [59]. The area of l. dorsi and fatness characteristics (% of pelvic and renal fat and subcutaneous fat depth) in this study were comparable to that found by Biçer et al. [60] in Awassi male lambs of similar CCWs. Leg tissue composition was also similar to that reported for Awassi 18 kg-weight carcasses [61].

Dietary treatments affected only CCW and CIC, with this effect being the consequence of the lowest values in both the ADG and final BW recorded in the LP lambs. No differences were observed between the MP and HP lambs in carcass characteristics despite the observed differences in ADG, which corroborates that increasing CP content beyond 15–16% does not substantively modify carcass characteristics in heavy fattening Assaf lambs, as has been reported in other sheep breeds [37,38].

The use of low protein diets in order to enhance the intramuscular fat content in ruminant meat has been evaluated, and results have been inconsistent [62]. In this trial, meat characteristics were not affected by dietary treatments, which would be in concordance with the above-mentioned lack of a substantively effect on carcass quality and with previous studies [19].

Discoloration and TBARS were also not affected by dietary treatments. This suggest that the level of protein in the diet for these animals would not have significantly modified the meat characteristics responsible for the myoglobin and lipid oxidative stability during storage [63].

As expected, intramuscular fat content, meat yellowness, and shear force values were greater and lightness values were lower than those reported for younger male fattening Assaf lambs [19,20]. It is well-known that as both age and BW at slaughter increase, meat becomes darker and redder as a consequence of a higher myoglobin concentration [64,65]. Meat yellowness has been positively related to carotene deposition but also with intramuscular fat deposition, which increases as animals age and carcass weights increase [64]. Nevertheless, Cohen-Zinder et al. [14] reported much lower meat yellowness values for heavier Assaf male lambs (64–65 kg of BW), which could have been due to the fact that these lambs showed a much higher ADG (on average > 400 g/day) and a lower meat fat content values than those recorded in our study. 

Changes in meat color during aerobic storage are mainly attributed to variations in oxymyoglobin concentration in the meat surface [66]. Both higher values for a* and a higher reflectance ratio Rλ_610_/Rλ_525_ are positively related to oxymyoglobin concentration in meat surfaces and denote higher meat redness [25]. In the present study, the results indicated a significant increase in redness (oxymyoglobin concentration in meat surface) from day zero to day two, when redness reached its maximum level, and then a subsequent decrease, with significant differences being observed between days 2 and 10 of storage. The higher redness at day two can be explained by the increase in oxymyoglobin concentration due to a loss of moisture from meat surface by water evaporation and a deeper oxygen penetration [67,68]. Afterwards, the decrease in the metmyoglobin-reducing capacity of meat resulted in the oxidation of metmyoglobin as the meat aged [69,70] in a quantity sufficient as to decrease redness, i.e., produce meat discoloration. The increase in metmyoglobin concentration in this study can be deduced from both the continuous increase, from day zero onwards, in Rλ_572_/Rλ_525_ and a decrease in Rλ_630_/Rλ_580_, which are, respectively, used for the calculation of the proportions of metmyoglobin and the sum of oxymyoglobin plus desoxymioglobin [25].

The changes in cooking losses (increased) and shear force (decreased) due to the five-day meat ageing period were expected and have been reported in other studies on lamb meat [71,72].

Regarding production costs, despite the beneficial effects of high dietary protein content on feed efficiency and growth performance, the low protein diet allowed us to reduce the feeding cost of carcass production (€/kg CCW). Therefore, reducing the protein supply could be an adequate strategy to improve the profitability of fattening lamb production and reduce the competency for nutritive resources with other livestock species that are more efficient than ruminants, which is an important challenge for ruminant production systems [9]. However, as male Assaf lambs may express higher growth rates than those recorded in the current study [14,19], further studies should be conducted to evaluate wider ranges of dietary protein content and slaughter weights.

## 5. Conclusions

Under the conditions of the present experiment, in which three groups of heavy fattening Assaf lambs were fed diets with different protein levels (134, 156, and 173 g CP/kg DM), the results suggest that the growth rate and the feed conversion ratio improve as protein content increases. Nevertheless, a protein content beyond 156 g/kg DM does not substantially improve either carcass or meat characteristics, and it could worsen profitability.

## Figures and Tables

**Table 1:** Ingredients, nutritive value, and cost of the experimental diets (low (LP), medium (MP) and high (HP) protein diets).

	LP	MP	HP
Ingredients (g/kg)			
Barley	532	512	490
Corn	210	199	189
Soybean meal, 48%	58	86	115
Barley straw	150	150	150
Molasses	10	10	10
Urea	4.8	7.1	9.5
Soybean oil	6	6	6
Mineral–vitamin premix	25	25	25
Sodium bicarbonate	5	5	5
Nutritive value (g/kg DM)			
Dry matter (g/kg)	878	889	881
aNDFom	236	237	250
Crude protein	134	157	173
Potential RDP ^1^	117	148	168
Effective RDP ^2^	73	92	99
Fat	37.6	22.7	29.4
Ash	5.90	6.50	7.81
Metabolizable energy (MJ/kg DM)	11.9	11.8	12.0
Cost (€/kg DM)	0.368	0.371	0.382

^1^ Potential rumen-degradable protein, calculated as the sum of fractions a and b; ^2^ Effective rumen degradation of protein estimated at a rate of passage of 0.083 h^−1^. aNDFom: neutral detergent fiber expressed without residual ash.

**Table 2:** Mean values of dry matter intake (DMI), average daily gain, and feed efficiency of heavy Assaf lambs fed diets with low (LP), medium (MP), and high (HP) crude protein contents.

	LP	MP	HP	SED ^1^	*p* Value
DMI, g/day	1256	1401	1464	97.36	0.108
Average daily gain, g/day	221 ^a^	268 ^ab^	297 ^b^	24.55	0.015
Feed conversion ratio, g/g	5.82 ^b^	5.32 ^ab^	4.97 ^a^	0.284	0.021
Residual feed intake, g	14.35	5.72	−20.07	19.682	0.405
Final body weight, kg	45.0	48.5	50.7	2.62	0.094

^1^ SED: standard error of the difference. ^a,b^ Means with different superscripts are significantly different (*p* < 0.05).

**Table 3:** Mean values of pH and volatile fatty acid (VFA) and ammonia concentration in the rumen fluid and in vitro fermentation parameters (ammonia concentration and gas and VFA production) of heavy Assaf lambs fed diets with low (LP), medium (MP), and high (HP) crude protein contents. NDF: neutral detergent fiber.

	LP	MP	HP	SED ^1^	*p* Value
In vivo parameters					
pH	6.54	6.72	6.43	0.257	0.579
Ammonia-N, mg/L	114	123	114	19.3	0.886
VFA concentration, mmol/L	119	98	140	22.8	0.304
Acetate, %	55.8	53.9	58.7	2.02	0.161
Propionate, %	24.4	29.9	19.3	4.41	0.151
Butyrate, %	13.0	9.9	16.5	2.70	0.145
Branched fatty acids, %	5.53	4.35	3.75	0.914	0.296
Valerate and caproate, %	1.33	1.90	1.71	0.279	0.224
Acetate/Propionate	2.52	1.86	3.11	0.474	0.112
In vitro fermentation using starch as substrate					
pH	6.11	6.21	6.36	0.135	0.281
Gas production, mmol	2.79	2.59	2.02	0.375	0.201
Ammonia-N, mg/L	62.8 ^a^	71.2 ^a^	165.2 ^b^	25.79	0.035
VFA production, mmol	3.49	3.53	2.98	0.257	0.135
Acetate, %	48.8	49.7	46.9	2.09	0.437
Propionate, %	37.5	38.5	33.3	9.18	0.840
Butyrate, %	11.8	9.0	15.5	7.74	0.709
Branched fatty acids, %	1.41	1.20	2.57	0.673	0.168
Valerate and caproate, %	0.54 ^a^	1.52 ^b^	1.72 ^b^	0.380	0.043
Acetate/Propionate	1.61	1.38	1.47	0.399	0.897
In vitro fermentation using NDF from straw as substrate					
pH	6.93	6.98	6.71	0.093	0.053
Gas production, mmol	1.02	0.94	1.12	0.115	0.334
Ammonia-N, mg/L	146	136	209	33.2	0.251
VFA production, mmol	1.55 ^a^	1.54 ^a^	2.48 ^b^	0.300	0.029
Acetate	68.6	65.4	55.1	4.42	0.051
Propionate	21.6	23.6	31.1	5.29	0.259
Butyrate, %	5.63	6.54	9.13	1.455	0.125
Branched fatty acids, %	3.20	3.11	3.03	1.189	0.991
Valerate and caproate, %	0.97	1.45	1.62	0.261	0.185
Acetate/Propionate	3.39	2.99	1.87	0.775	0.223

^1^ SED: standard error of the difference. ^a,b^ Means with different superscripts are significantly different (*p* < 0.05).

**Table 4:** Blood acid–base status and plasma biochemical profile of heavy Assaf lambs fed diets with low (LP), medium (MP), and high (HP) crude protein contents. pCO_2_: CO_2_ pressure; tCO_2_: total CO_2_; AST: aspartate aminotransferase; and ALT: alanine aminotransferase.

	Dietary Treatments			RSD ^1^	Sampling Days		RSD ^2^	*p* Values		
	LP	MP	HP		35	70		Diet	Day	Diet * Day
Acid–base status										
pH	7.43	7.43	7.43	0.032	7.44	7.43	0.033	0.938	0.191	0.421
HCO_3_^−^, mmol/L	27.6	26.2	26.9	1.66	27.6	26.2	1.06	0.215	<0.001	0.971
pCO_2_, mmHg	44.7	42.9	44.0	4.54	44.6	43.1	3.70	0.675	0.137	0.444
Anion gap, mmol/L	15.5	15.6	15.3	1.58	14.6	16.3	2.23	0.922	0.004	0.555
tCO_2_, mmol/L	28.9	27.5	28.3	1.76	29.0	27.5	1.10	0.228	<0.001	0.955
Na, mmol/L	148	147	147	1.5	148	148	1.9	0.336	0.047	0.680
K, mmol/K	5.38	5.25	5.53	0.802	5.73	5.05	0.645	0.740	<0.001	0.801
Cl, mmol/L	110	110	111	1.9	111	109	2.2	0.461	0.009	0.341
Biochemical profile										
Urea, mg/dL	33.3 ^a^	41.3 ^b^	36.9 ^ab^	6.01	34.8	39.6	5.98	0.016	0.005	0.335
Protein, g/L	59.5 ^a^	61.8 ^ab^	63.5 ^b^	2.90	59.3	63.9	3.50	0.018	<0.001	0.895
Albumin, g/L	35.2 ^a^	37.8 ^b^	38.1 ^b^	2.08	35.8	38.2	2.30	0.010	<0.001	0.121
ALT, U/L	15.4	17.6	18.0	2.82	17.7	16.2	1.91	0.102	0.006	0.435
AST, U/L	96.9	94.0	92.1	17.04	91.6	97.1	14.88	0.813	0.163	0.355
Creatinine, mg/dL	1.11	1.10	1.12	0.085	1.07	1.15	0.062	0.745	<0.001	0.222
Glucose, mg/dL	101 ^a^	108 ^b^	111 ^b^	5.3	105	109	6.4	0.001	0.015	0.051
Lactate, mg/dL	21.6	19.7	17.6	5.55	18.5	20.7	9.28	0.435	0.276	0.576
Ca, mg/dL	11.2 ^a^	11.6 ^ab^	11.8 ^b^	0.47	11.1	12.0	0.61	0.019	<0.001	0.066
P, mg/dL	8.77	8.80	9.02	0.905	8.58	9.15	0.224	0.776	0.018	0.327
Cholesterol, mg/dL	68.4	62.8	73.2	10.28	66.4	69.9	7.27	0.104	0.073	0.144
Triglycerides, mmol/L	33.8	33.7	37.8	6.03	45.2	25.1	5.97	0.223	<0.001	0.232

^1^ RSD: residual standard deviation to compare dietary treatments; ^2^ RSD: residual standard deviation to compare days; ^a,b^ Means with different superscripts are significantly different (*p* < 0.05).

**Table 5:** Carcass characteristics of heavy Assaf lambs fed diets with low (LP), medium (MP), and high (HP) crude protein contents. L: carcass internal length; F: pelvic limb length; CIC: compactness index of carcass.

	LP	MP	HP	SED ^1^	*p* Value
Cold carcass weight (CCW), kg ^2^	22.8	24.7	26.1	1.50	0.117
Dressing percentage, %	50.6	50.7	51.1	0.83	0.809
Chilling losses, %	2.43	1.77	1.84	0.296	0.067
pH at 24 h	5.71	5.82	5.72	0.052	0.065
Pelvic and renal fat, % of CCW	2.26	2.07	2.56	0.304	0.280
Subcutaneous fat depth, mm ^2^	7.2	10.0	8.9	2.45	0.280
L, cm	63.8	65.6	65.9	1.29	0.140
F, cm	42.2	42.8	42.8	0.68	0.426
TH, cm	27.9	28.5	28.3	0.51	0.413
CIC, g/cm	334	356	385	25.9	0.160
Subcutaneous fat color					
L*	68.5	69.6	68.0	1.51	0.572
a*	1.88	2.24	2.79	0.459	0.154
b*	8.11	7.69	8.19	0.749	0.772
Commercial cuts, % ^3^					
Higher priced	52.9	52.6	52.6	0.47	0.674
Medium priced	18.2	18.3	18.2	0.36	0.943
Lower priced	26.5	27.0	26.6	0.464	0.516
Longissimus dorsi characteristics					
Total weight, % ^4^	4.91	4.80	4.80	0.24	0.752
Area, square cm ^5^	15.9	15.8	15.3	0.21	0.903
Leg tissue composition, %					
Muscle	60.1	59.6	60.4	1.31	0.704
Fat	15.9	17.3	17.5	1.48	0.279
Bone	22.0	21.2	20.8	0.82	0.125
Remainders	2.05	1.91	1.40	0.377	0.053

^1^ SED: Standard error of the difference. ^2^ Depth of the layer of subcutaneous fat covering the loin at the level of the 13th rib. ^3^ Legs, loin, and foreribs comprised the higher priced joints; shoulders comprised the medium price joints; and the lower priced joints included breast, neck, and tail. ^4^ Percentage of loin in the sum of commercial cuts. ^5^ Area calculated from the semi-major (width) and semi-minor (depth) l. dorsi axes at the level of the 13th rib.

**Table 6:** Meat quality traits (longissimus muscle) in the heavy Assaf lambs fed diets with low (LP), medium (MP), and high (HP) crude protein contents. TBARS: thiobarbituric acid reactive substances.

	Dietary Treatments			RSD ^1^	Storage Time (Days)				RSD ^2^	*p* Value		
	LP	MP	HP		0	2	5	10		Diet	Day	Diet * Day
L. thoracis, raw												
Composition, g/kg												
Water	769	764	761	9.6	-	-	-	-	-	0.192	-	-
Protein	191	196	195	6.7	-	-	-	-	-	0.215	-	-
Intramuscular fat	28.6	28.9	29.4	8.0	-	-	-	-	-	0.974	-	-
Ash	11.3	11.7	11.8	1.1	-	-	-	-	-	0.592	-	-
Color and color stability												
L*	39.0	38.0	38.2	1.31	37.6	38.6	38.8	38.6	2.46	0.060	0.160	0.996
a*	8.19	8.26	8.50	0.921	7.41 ^c^	9.10 ^a^	8.63 ^ab^	8.12 ^bc^	1.74	0.412	0.001	0.799
b*	10.3	9.8	10.5	1.13	8.3 ^c^	11.4 ^a^	10.5 ^a^	10.7 ^a^	1.96	0.134	0.001	0.644
a*/b*	0.81	0.87	0.83	0.347	0.916 ^a^	0.819 ^ab^	0.840 ^ab^	0.778 ^b^	0.624	0.185	0.003	0.952
Rλ_610_/Rλ_525_^4^	1.76	1.77	1.78	0.324	1.69 ^c^	1.89 ^a^	1.78 ^b^	1.75 ^bc^	0.600	0.845	0.001	0.872
Rλ_630_/Rλ_580_^4^	2.47	2.47	2.45	0.418	2.95 ^a^	2.69 ^b^	2.20 ^c^	2.01 ^d^	0.774	0.914	0.001	0.189
Rλ_572_/Rλ_525_^4^	0.879	0.877	0.874	0.199	0.748 ^d^	0.857 ^c^	0.927 ^b^	0.975 ^a^	0.368	0.907	0.001	0.925
L. lumborum, cooked												
Cooking losses, %	21.4	21.6	21.3	3.91	19.9		22.9	-	2.94	0.986	0.001	0.515
Shear force, N	93.8	89.4	81.9	17.8	92.8		83.9	-	9.38	0.339	0.001	0.834
L. thoracis, cooked												
∆TBARS, mg MDA/kg ^3^	2.68	3.12	3.34	0.953	-	-		-	-	0.306	-	-

^1^ RSD: residual standard deviation to compare dietary treatments; ^2^ RSD: residual standard deviation to compare days of storage; ^3^ Increment in thiobarbituric acid reactive substances during 2 days of refrigerated storage; MDA: malondialdehyde; ^a,b^ Means with different superscripts are significantly different (*p* < 0.05). ^4^ Reflectance ratios
